# *Astragalus* polysaccharides-induced gut microbiota play a predominant role in enhancing of intestinal barrier function of broiler chickens

**DOI:** 10.1186/s40104-024-01060-1

**Published:** 2024-08-06

**Authors:** Jiantao Yang, Yanpeng Sun, Qianggang Wang, Shanglin Yu, Yanhe Li, Bin Yao, Xiaojun Yang

**Affiliations:** 1 College of Animal Science and Technology, Northwest A&F University, Yangling, Shaanxi China; 2 State Key Laboratory of Animal Nutrition, Institute of Animal Sciences, Chinese Academy of Agriculture Science, Beijing, China

**Keywords:** *Astragalus* polysaccharides, Broiler, Gut microbiota, Intestinal barrier function

## Abstract

**Background:**

The intestinal barrier is the first line of defense against intestinal invasion by pathogens and foreign antigens and is closely associated with the gut microbiota. *Astragalus* polysaccharides (APS) have a long history of use in traditional Chinese medicine owing to its protective properties against intestinal barrier function. The mechanism of APS-induced gut microbiota enhancing intestinal barrier function is urgently needed.

**Results:**

Dietary polysaccharide deprivation induced intestinal barrier dysfunction, decreased growth performance, altered microbial composition (*Faecalibacterium*, *Dorea*, and *Coprobacillus*), and reduced isobutyrate concentration. The results showed that APS facilitates intestinal barrier function in broiler chickens, including a thicker mucus layer, reduced crypt depth, and the growth of tight junction proteins. We studied the landscape of APS-induced gut microbiota and found that APS selectively promoted the growth of *Parabacteroides*, a commensal bacterium that plays a predominant role in enhancing intestinal barrier function. An in vitro growth assay further verified that APS selectively increased the abundance of *Parabacteroides distasonis* and *Bacteroides uniformis.* Dietary APS supplementation increased the concentrations of isobutyrate and bile acid (mainly chenodeoxycholic acid and deoxycholate acid) and activated signaling pathways related to intestinal barrier function (such as protein processing in the endoplasmic reticulum, tight junctions, and adherens junction signaling pathways).

**Conclusions:**

APS intervention restored the dietary polysaccharide-induced dysfunction of the intestinal barrier by selectively promoting the abundance of *Parabacteroides distasonis,* and increasing the concentrations of isobutyrate and bile acids (mainly CDCA and DCA). These findings suggest that APS-induced gut microbiota and metabolic niches are promising strategies for enhancing intestinal barrier function.

**Graphical Abstract:**

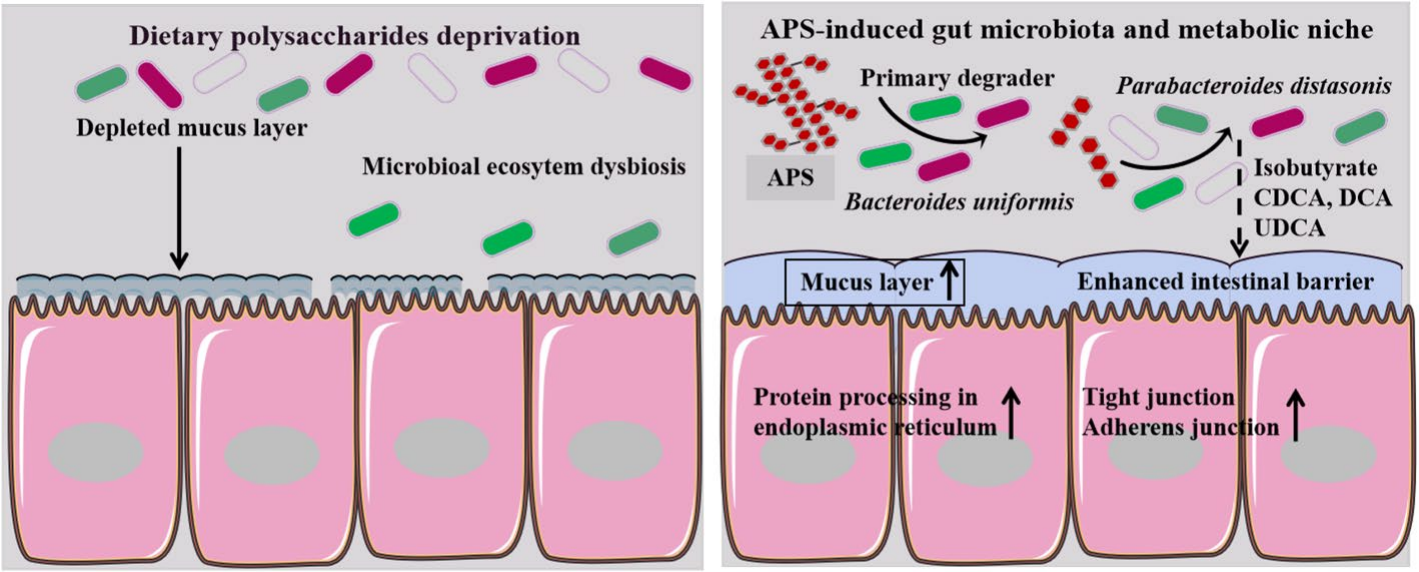

**Supplementary Information:**

The online version contains supplementary material available at 10.1186/s40104-024-01060-1.

## Background

Global consumption of chicken meat has shown a soaring trend owing to intensive selection and growth rate with efficient feed conversion ratios of broilers [1, 2]. It is estimated that the number of live chickens worldwide reached 23 billion, and chicken became the largest meat producer worldwide in 2019 [3]. However, crowded breeding conditions and various environmental stressors increase the risk of disease in poultry and can cause significant economic losses in the poultry industry [4]. Particularly, in the early stages of life, the immune system of broilers shows low immune activity [5]. It has been suggested that the intestine is an important organ for food digestion and nutrient absorption and plays a key role in immune regulation [6]. In poultry production, the intestinal barrier may be impaired by diet and environmental stressors such as mycotoxins, pathogens, and heat stress [7, 8]. Dysfunction of the intestinal barrier increases the selective permeability of the intestinal mucus barrier, causing decreased thickness of the mucus layer and increased translocation of pathogens. It is also associated with systemic inflammatory responses, celiac disease, and metabolic diseases [9–11]. The intestinal mucus barrier, which is located at the interface of the gut microbiota and intestinal epithelium, is considered a crucial intestinal barrier in the physiological defense against mechanical and chemical attacks [12, 13]. Mucin secreted by goblet cells forms the structural framework of the intestinal mucus barrier and is associated with intestinal barrier function [14, 15]. The early stages of life are key physiological periods for mucin secretion and mucus intestinal layer in broilers [16]. In general, improving the intestinal barrier function in early poultry breeding is crucial from an economic perspective.

Trillions of microbiota inhabit the intestinal tract, and the gut microbiota is a complex and dynamic microbial ecosystem [17, 18]. There is compelling and converging evidence suggesting that the gut microbiota and its derived bioactive substances play a causative role in maintaining intestinal barrier function [9, 19]. For example, the gut commensal *Bacteroides thetaiotaomicron*-derived acetate promotes intestinal mucin synthesis and glycosylation via the transcription factor Kruppel-like factor 4 (KLF4), which is involved in goblet cell differentiation [20]. Another notable example is the production of short-chain fatty acids (SCFAs) through the microbial fermentation of non-digestible carbohydrates, which provide an energy source for epithelial cells and protect the intestinal mucus barrier [21–23]. The gut microbiota is involved in bile acid biotransformation, and chenodeoxycholic acid (CDCA) alleviates lipopolysaccharide-induced intestinal barrier impairment [24]. Therefore, deciphering the underlying relationship between the gut microbiota, intestinal barrier function, and manipulation of the microbial community provides a unique opportunity to improve intestinal barrier function through microbes and their derived metabolites.

*Astragalus* polysaccharides (APS) are large molecular weight polymers with a molecular weight range of 8.7–4,800 kDa, which are isolated and purified from the root of *Astragalus membranaceus* [25, 26]*.* Increasing experimental evidence has demonstrated that APS has various biological activities such as antidiabetic, anti-inflammatory, antioxidant, and immunomodulatory activities [27, 28]. Recent studies have shown that the degradation of plant polysaccharides requires the involvement of carbohydrate enzymes, and that polysaccharides act as fuel for the gut microbiota to shape the microbial community [29–31]. For example, arabinoxylan and resistant starch maintain intestinal mucus barrier function by selectively targeting *Blautia*, *Ruminococcus*, and *Bacteroides* in broilers [12]. However, the causative relationship between APS and the microbiota in the intestinal barrier remains elusive. Whether APS can act as a nutrient substrate for gut microbiota, thereby improving the intestinal mucosal barrier by forming an exclusive microbial metabolic niche, is urgently needed.

In the present study, we hypothesized that APS could manipulate the gut microbiota and microbial metabolic pathways, thereby maintaining the intestinal barrier function of broilers. To further understand and systematically decipher the beneficial effects of APS on the intestinal barrier function and gut microbiota of broilers, we used a polysaccharide-deprived diet to construct a gut dysbiosis model. An in vitro growth assay further explored microbiota-associated mechanisms. This study revealed the existence of APS-induced commensal microbiota responsible for the protection of intestinal barrier function in broilers, providing a basis for future nutritional interventions to improve intestinal barrier function.

## Materials and methods

### Preparation and separation of *Astragalus* polysaccharides

APS was isolated and extracted as described previously [26]. Briefly, the powder of *Astragalus mongholicus* Bunge was passed through a 60-mesh sieve. Deionized water added to the powder of *Astragalus mongholicus* Bunge at a ratio of 1:20 (w/v), and the mixture was incubated for 4 h at 90 °C for extraction. The absolute ethanol was added to the extraction to achieve a final alcohol concentration of 90%. Next, 5% trichloroacetic acid was added to extraction at a ratio of 1:1 (v/v), and the mixture stand at 4 °C for 24 h. The mixture was centrifuged at 4,500 × *g* for 10 min at 4 °C to remove proteins. The supernatant was freeze-dried to obtain APS. In the present study, the phenol-sulfuric acid method was used to determine the purity. The calculated total polysaccharide content is 80.21g/100g. The protein content was measured by using Coomassie brilliant blue method. The calculated protein content is 0.29%. Also, the monosaccharide composition and molecular weight distribution of APS was determined by high-performance gel filtration chromatography (HPGFC), and the results as Table 1.

**Table 1:** Molecular weight analysis of APS subfractions

Subfraction	Component	Molecular weight (MW)	Percentage, %
G1	Polysaccharide	26–60 kDa	64.7
G2	Polysaccharide	60–500 kda	30.8
G3	Polysaccharide	> 500 kda	4.5

### Animals and diets

The approval for all the procedures related to animal’s experimentation was obtained from the Animal Care and Use Committee of the College of Animal Science and Technology of the Northwest A&F University (Shaanxi, China), and all operations were conducted according to the university’s guidelines for animal research.

**Fig. 1:**
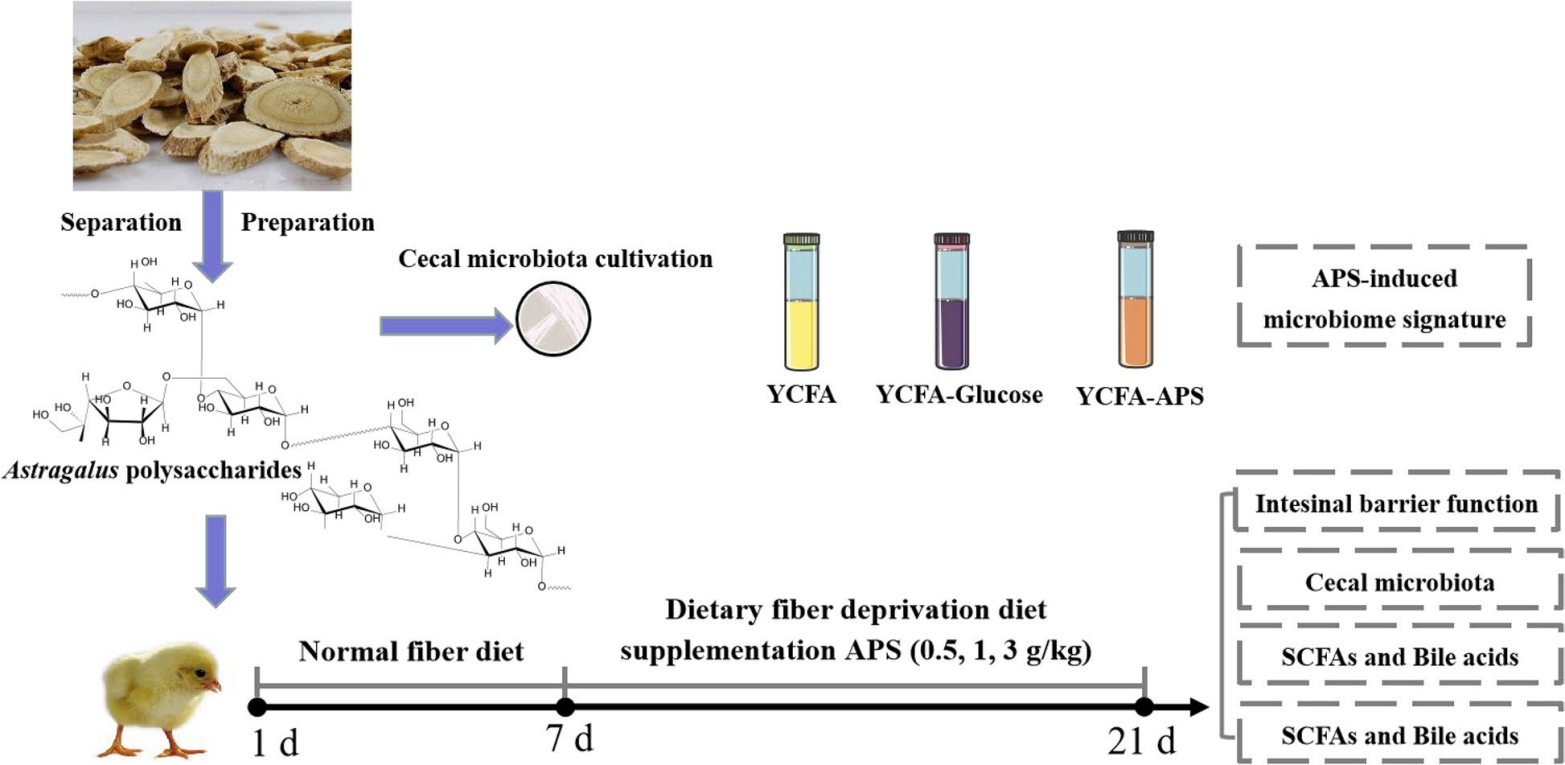
Overview of experiment design

In the study, a total of 200 one-day-old Arbor Acres broilers were selected and randomly divided into 5 groups as follows: CON (normal fiber level groups), NC (dietary polysaccharides deprivation diet groups), APSI (dietary polysaccharides deprivation diet with APS at 5 g/kg), APSII (dietary polysaccharides deprivation diet with APS at 10 g/kg), and APSIII (dietary polysaccharides deprivation diet with APS at 30 g/kg). Each group contained 10 replicates with 4 birds per replicate. As shown in Fig. 1, after an accommodation period of 1 week, all broilers were fed a standard corn-soybean meal diet. Then, the diets were arranged to one of five dietary as follows for 8–21 d: (1) standard corn-soybean meal diet (CON), (2) NC, (3) APSI, (4) APSII, and (5) APSIII. The ingredients and nutrient levels of the basal diets are shown in Table 2. All birds were fed in double-layer wired battery cages with ad libitum access to water and mash feed at the Experimental Teaching Center of Animal Science in the Norwest A&F University. Animals were housed in an environmentally-controlled room with temperatures starting at 35 °C, and then decreased by 2 °C every 7 d. At 21 d, feed intake, body weight, average daily gain and feed conversion ratio were recorded and calculated for each replicate. At the end of the experiment, one male bird was selected from each replicate and serum samples were collected by centrifuging venous blood at 2,000 × *g* for 15 min at 4 °C, and then euthanized by CO_2_ asphyxiation. Approximately 0.7 g of the ileal and cecal contents and feces were collected from each bird. In addition, the ileum resected and washed with cold PBS were opened longitudinally and the mucosa was collected. The 0.5 g of the liver was collected. All samples were frozen immediately in liquid nitrogen, and then stored at −80 °C.

**Table 2:** Analyzed nutrient composition of experiment diets (as-fed basis)

Ingredients, %	CON	NC	APSI	APSII	APSIII
Corn	57.27	0.00	0.00	0.00	0.00
Corn starch	0.00	58.25	55.25	55.25	55.25
Soybean meal	35.2	0.00	0.00	0.00	0.00
Soy protein concentrate	0.00	32.68	32.68	32.68	32.68
Cottonseed meal	2.00	0.00	0.00	0.00	0.00
Soybean oil	2.00	3.52	3.52	3.52	3.52
Sucrose	0.00	0.50	0.50	0.50	0.50
APS	0.00	0.00	0.5	1.00	3.00
NaCl	0.36	0.31	0.31	0.31	0.31
Limestone	2.00	0.80	0.80	0.80	0.80
Calcium hydrogen phosphate	0.30	3.32	3.32	3.32	3.32
Choline chloride	0.05	0.10	0.10	0.10	0.10
L-Lysine hydrochloride	0.14	0.00	0.00	0.00	0.00
Mineral premix^a^	0.30	0.10	0.10	0.10	0.10
Phytase	0.10	0.00	0.00	0.00	0.00
Vitamin premix^b^	0.03	0.03	0.03	0.03	0.03
DL-Methionine	0.25	0.16	0.16	0.16	0.16
Sodium bicarbonate	0.00	0.20	0.20	0.20	0.20
Antioxidant	0.00	0.03	0.03	0.03	0.03
Total	100	100	100	100	100
Calculated nutrient level					
Metabolic energy, kcal/kg	2,950	2,960	2,866	2,866	2,866
Dry matter, %	86.41	95	95	95	95
Crude fiber, %	3.02	1.67	1.67	1.67	1.67
Crude protein, %	21.91	21.00	21.00	21.00	21.00
Ash, %	6.26	6.42	6.42	6.42	6.42
Total phosphorus, %	0.61	0.61	0.61	0.61	0.61
Calcium, %	0.96	0.96	0.96	0.96	0.96
Methionine, %	0.57	0.45	0.45	0.45	0.45
Lysine, %	1.21	1.38	1.38	1.38	1.38

^a^Mineral premix provided the following per kg of the diet: Mn, 80 mg; I, 0.40 mg; Fe, 80 mg; Cu, 10 mg; Zn, 70 mg; Se, 0.30 mg

^b^Vitamin premix provided the following per kg of the diet: vitamin A, 250,000 IU; vitamin D, 50,000 IU; vitamin K_3_, 53 mg; vitamin B_1_, 40 mg; vitamin B_2_, 120 mg; vitamin B_12_, 0.50 mg; vitamin E, 600 IU; biotin, 0.65 mg; folic acid, 25 mg; pantothenic acid, 240 mg; niacin, 1,000 mg

### Intestinal morphology analysis

For morphology, proximal ileal tissues were freshly harvested from broilers and fixed with 4% formaldehyde over 48 h prior to paraffin embedding for hematoxylin-eosin (H&E) analysis. Morphological changes were examined under a light microscope (Olympus Corporation, Tokyo, Japan) coupled with image processing software (Image J 1.53). For analysis, five crypts and villi were randomly selected from different parts of the sample and measured using Image J 1.53.

The number of ileal mucin was measured as previously described [12]. Briefly, Carnoy’s fixed ileal tissues were stained with the Alcian blue-periodic acid Schiff (AB-PAS) and periodic acid Schiff (PAS) following the manufacture’s instruction (Servicebio, Wuhan, China). The stained slides were measured with light microscope (Olympus Corporation, Tokyo, Japan). The counts of mucin found in 5 randomly chosen intact villus per section.

### Measurement of ileum mucous layer thickness

Thickness of the ileal mucus layer was measured as described [32]. The proximal ileal tissues fixed in Carnoy’s solution for 4 h followed by transfer to fresh Carnoy’s solution for 4 h. The ileal tissues were then washed in cold dry methanol twice for 2 h, stored in fresh dry methanol at 4 °C until further use. Post Carnoy’s fixation, ileal tissues were embedded in paraffin and thin sections (~5 μm) were cut. Alcian blue staining was performed by the following protocol: 1) deparaffinization at 65 °C for over 4 h, 2) dehydration for 2 min with different alcohol concentration gradients (100%, 95%, 90%, 80%, 70%), 3) Alcian blue solution for 25 min, 4) washing in running tap water for 1 min, 5) dehydration for 4 min with 95% and absolute alcohol, and 6) cleanout in xylene and cover with coverslip. To measure the thickness of ileal mucus layer, 5 intact mucus layer units were selected using a light microscope (Olympus Corporation, Tokyo, Japan) coupled with image processing software (Image J 1.53).

### In vivo intestinal permeability analysis assay using FITC-dextran

For the fluorescein isothiocyanate (FITC)-dextran (4 kDa; Sigma) assay, chickens were fasted for 6 h and then were orally gavaged with FITC-dextran (7 mg/kg body weight) 3 h before blood collection. The concentration of FITC was monitored using a fluorescence spectrophotometer with an excitation wavelength of 493 nm and an emission wavelength of 525 nm.

### Measurement of plasma diamine oxidase activities, and lactic acid levels

The plasma diamine oxidase and lactic acid levels were measured using enzyme-linked immunosorbent assay (ELISA; Nanjing Jiancheng Bioengineering Institute Nangjing, China).

### RNA isolation and quantitative real-time PCR (qRT-PCR)

Detail PCR reaction and calculation methods were performed as previously description [33]. The ileal mucosa was homogenized and total RNA was extracted according to TRIzol reagent protocol (AG21102, AG, Changsha, China). RNA concentration and purity were measured using NanoDrop 2000. cDNA was synthesized from 500 ng RNA using Evo M-MLV Reverse Transcriptase Kit (AG11707, AG, Changsha, China). The mRNA expression was performed using the SYBR Green Premix Pro Taq HS qPCR Kit (AG11701, AG, Changsha, China) on the iCycler IQ5 (Bio-Rad, Hercules, CA, USA). All primer sequences in the study are listed in Table 3 as previously description [33].

**Table 3:** Forward and reverse primer sequences for PCR analysis

Genes	GenBank accession number	Forward primer (5′→3′)	Reverse primer (5′→3′)	Amplicon size, bp
*β-actin*	L08165	ATTGTCCACGCAAATGCTTC	AAATAAAGCCATGCCAACTCGTC	173
*MUC2*	XM_040673055.2	TTCATGATGCCTGCTCTTGTG	CCTGAGCCTTGGTACATTCTTG	93
*ZO-1*	XM_413773	GGGATGTTTATTTGGGCGGC	TCACCGTGTGTTGTTCCCAT	187
*Occludin*	NM_205128.1	TCATCGCCTCCATCGTCTAC	TCTTACTGCGCGTCTTCTGG	240
*Claudin*	NM_001013611	ACCCACAGCCTAAGTGCTTC	AGGTCTCATAAGGCCCCACT	200

Abbreviations: *β-actin* The internal reference gene beta actin, *MUC2* Mucin 2, *ZO-1* Zonula occludens 1

### 16S rDNA gene amplicon sequencing and data analysis

The E.Z.N.A. soil DNA Kit (Omega Bio-tek, Norcross, GA, USA) was used to extracted bacterial genomic DNA from ileal contents according to manufacturer’s protocols. The bacterial V3-V4 region was amplified using the following primers: 338F (5′-ACTCCTACGGGAGGCAGCAG-3′) and reverse primer 806R (5′-GGACTACHVGGGTWTCTAAT-3′). The PCR amplification products was sequenced on the Illumina MiSeq platform (Illumina, San Diego, USA) according to the standard protocols. Sequence raw data were analyzed with QIIME2 platform. Quality control and denoising were conducted using DADA2 with default parameters to generate ASVs. The principal coordinate analysis (PCoA) based on Bray-Curtis distance, α-diversity and LEfSe (liner discriminant analysis effect size) analysis were conducted on the free online platform of Microeco Tech Co., Ltd. (Shenzhen, China, https://bioincloud.tech/task-list). The co-occurrence network analysis was conducted Spearman’s rank correlation coefficient based on the relative abundance profile of genera and networks were then constructed by using the method implemented in Cytoscape. Statistical differential taxa were evaluated using the non-parametric Kruskal-Wallis test with false discovery rate (FDR) correction for multiple testing (*P *< 0.05).

### Short-chain fatty acids (SCFAs) profiling in cecum

Detailed analytical methods of SCFAs were set as reported methods [34]. In brief, the concentration of SCFA in the cecal contents were measured using gas chromatography-mass spectrometry (GC-MS). Samples were thawed on ice and approximately 0.3 g cecal contents were homogenized with 1 mL of cold normal saline and centrifuged at 4 °C at 13,000 × *g* for 10 min. The supernatant was diluted with 0.4 mL metaphosphoric acid for deproteinization. After 4 h quiescence at 4 °C, the mixture was centrifuged at 4 °C at 13,000 × *g* for 10 min. The supernatant was mixed with 0.2 mL crotonic acid. The mixture was filtered through a 0.45-μm filter. Finally, the extracted sample solution was measured by GC-MS on an Agilent 6890 (Agilent Technologies, CA, USA).

To determine the SCFAs concentration of the bacteria medium, 1 mL medium was obtained and centrifuged at 4 °C at 13,000 × *g* for 10 min. The rest of the procedures were same as above.

### Bile acids quantification in plasma, liver and ileum sample

The bile acid analysis from plasma, liver, and contents of ileum were performed using liquid chromatography-mass spectrometry (LC-MS) as previously described [35]. Briefly, 0.2 mL plasma, approximately 60 mg liver and ileal contents were extracted with 0.6 mL methanol:water (1:1) extract. After 15 min of vortex, and then 15 min of centrifugation at 13,000 ×* g* at 4 °C, followed the supernatant was mixed with methanol:acetonitrile (2:8). The homogenized solution was vortex for 15 min, and then centrifuged at 13,000 × *g* at 4 °C for 15 min. The supernatant was filtered using 0.22-μm filter. Finally, the LC-MS (Qtrap 5500, AB SCIEX) analysis was performed. The standard curves were made for quantification.

### In vitro simulated digestion of APS

Prepare the saliva digestive solution by combining 1.126 g/L of KCl, 1.144 g/L of NaHCO_3_, 0.12 g/L of NaCl, 0.168 g/L of CaCl_2_, and 150 U/L of α-amylase. For the gastric digestive solution, mix 1.1 g/L of KCl, 3.1 g/L of NaCl, 0.6 g/L of NaHCO_3_, 0.15 g/L of CaCl_2_, and 0.24 g/L of pepsin, 0.254 g/L of gastric lipase and 0.02 mol/L of CH_3_COONa, and adjust the pH to 2.0 with hydrochloric acid (0.1 mol/L). Lastly, prepare the intestinal digestive solution with 0.65 g/L of KCl, 5.4 g/L of NaCl, 0.33 g/L of CaCl_2_, 0.13 g/L of trypsin, 0.07 g/L of pancreatin, and 0.016 g/L of bile salt. The above 3 solutions are prepared separately. Then, the simulated digestive solution was prepared by mixing 5 mL APS aqueous solution (1 g/L) with 5 mL salivary digestive solution, dissolving 0.025 g APS in 25 mL gastric digestive solution, and after 4 h of gastric digestion, mixing the gastric digestive solution that was adjusted the pH to 7 with NaHCO_3_ (1 mol/L) and intestinal digestive solution at the ratio of 10:3. All simulated digestive mixtures were incubated in an oscillating water bath at 37 °C. The 1 mL mixture was taken out from simulated salivary mixture at 0, 0.5, 1, and 2 h, and was taken out from simulated gastric, and intestinal mixture of 1 mL at 0, 1, 2, and 4 h, respectively. Then, the extracted mixture was boiled at 100 °C for 10 min to eliminate enzyme activity. Prepare glucose standard solution (0, 0.2, 0.4, 0.6, 0.8, 1.0 mg/mL of glucose) and determine the standard curve by enzyme reader according to phenol sulfuric acid method and 3,5-dinitrosalicylic acid method. The absorbance value of the boiled mixture was measured by phenol sulfate method and 3,5-Dinitrosalicylic acid method to calculate whether the total sugar and reducing sugar changed according to the standard curve.

### In vitro anaerobic culturing of APS-induced gut microbiota

Fresh cecal content from five healthy male 21-day-old broilers immediately transferred into an anaerobic chamber. The approximately 1 g cecal content was added to 10 mL sterile Ringer working buffer (0.4 g/L of potassium chloride, 9 g/L of sodium chloride, 0.25 g/L of calcium dehydrate and 0.05% (w/v) L-cysteine hydrochloride) and vortexed for 5 min. Then, the mixture was filtered through three layers of cheesecloth. The mixture was centrifuged at 2,000 × *g* at 4 °C for 5 min, bacteria-enriched supernatants were obtained. The 1 mL supernatants immediately transferred into 10 mL YCFA medium containing 1% (w/v) glucose or APS, and the medium without any carbon source served as negative control (Con). Cultures (15 mL) were grown under anaerobic conditions at 37 °C. Cultures were collected at 4, 8, 12, 16, 20, 24, 28, 32, 36, 40, 44, and 48 h for OD_600_, pH, lactic acid, SCFAs and 16S rDNA analysis.

### RNA sequencing

The ileal mucosa was collected and the total RNA was extracted by using TRIzol reagent. RNA purity was evaluated by the Nanophotometer spectrophotometer (IMPLEN, CA, USA). Samples with RNA integrity was assessed using the RNA Nano 6000 Assay Kit and with 260/280 nm absorbance ratios from 1.9 to 2.1 were used for the construction of RNA-Seq libraries. Libraries were generated using NEBNext^®^ Ultra^TM^ RNA Library Prep Kit for Illumina^®^ (NEB, USA) according to the manufacturer’s instructions. Sequencing of the libraries was performed on an Illumina HiSeq2000 platform, and Raw data assessed for quality using FastQC. Analysis of differential expression was performed using DESeq2 R package. The resulting *P*-values were adjusted according the Benjamini and Hochberg’s approach. Genes with a corrected *P*-value < 0.05 and fold changes > 2 were assigned as differentially expressed.

### Statistical analysis

The software GraphPad Prism V8 was used for statistical analysis. The Tukey’s analysis was used for parametric ANOVA between groups. The original FDR method of Benjamini and Hocheberg was used for non-parametric ANOVA between groups. Data are presented as mean ± SEM. Statistical significance was set at *P *< 0.05, which is indicated as follows: ^*^P < 0.05, ^**^P < 0.01.

## Results

### Characterization of APS from *Astragalus membranaceus* extract

**Fig. 2:**
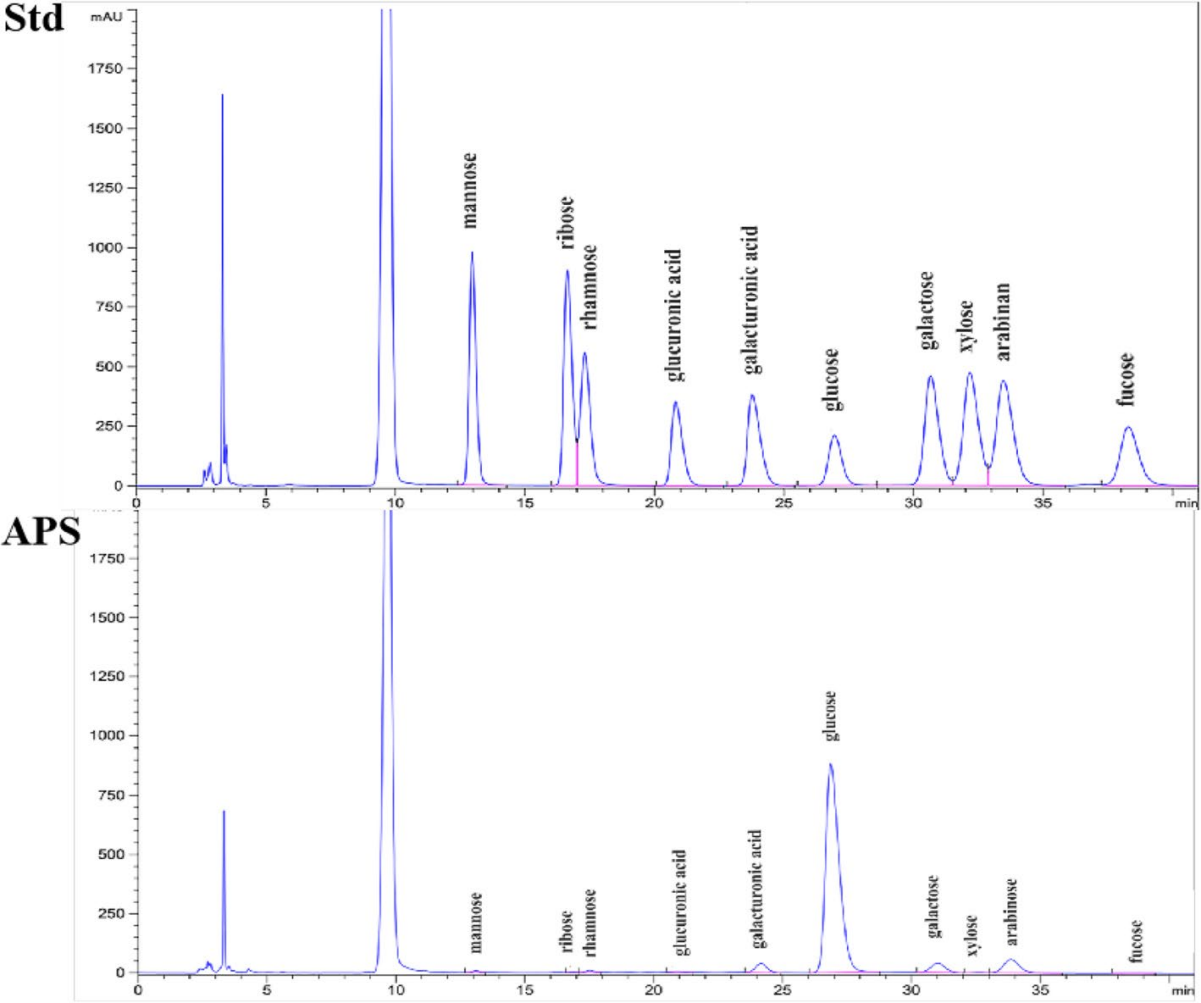
Monosaccharide composition of APS. Std, Standard

Our published data show that the structural features, molecular weight, and glycosidic bonds of APS are related biological properties [36]. In the present study, APS (61.74% polysaccharide; crude protein at 0.298 g/100 g) was prepared from the roots of *Astragalus membranaceus* using a water extract with ethanol. APS, as shown in Fig. 2, is a heteropolysaccharide composed mainly of mannose, glucose, rhamnose, glucuronic acid, galacturonic acid, galactose, arabinose, and fucose. The molecular weight of APS was 64.7% in the 26–60 kDa, 30.8% in the 60–500 kDa, and 4.5% in the > 500 kDa range (Table 1).

### Growth performance

**Fig. 3:**
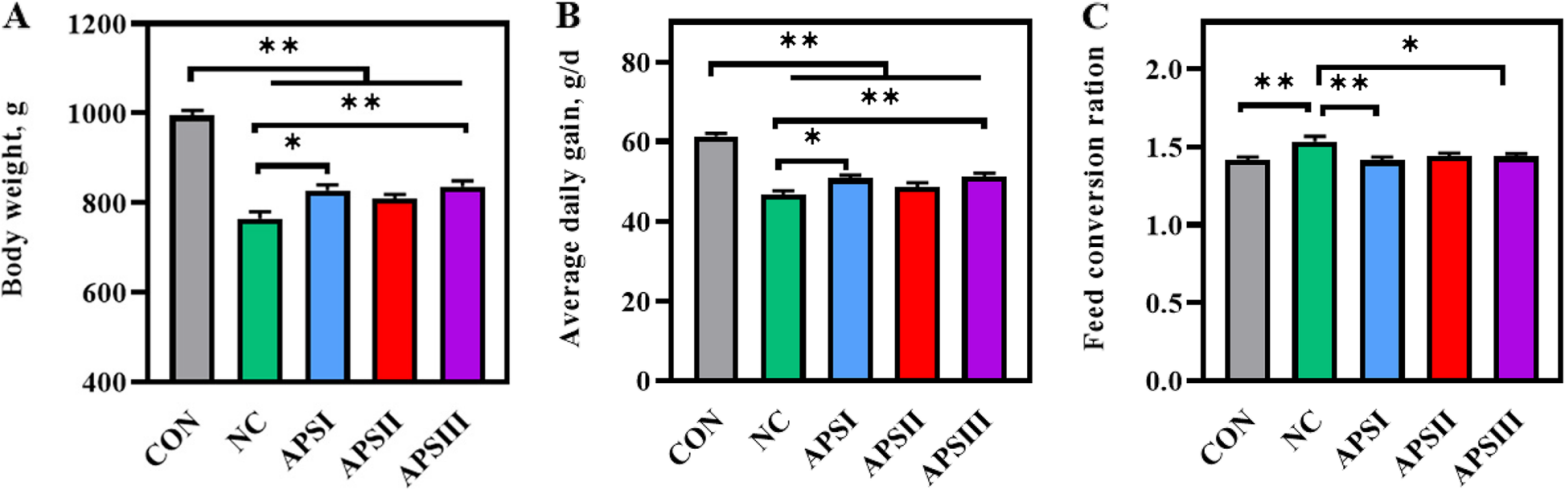
Effects of APS supplementation on growth performance of broilers at 21 d. **A** Body weight. **B** Average daily gain. **C** Feed conversion ration. All data are expressed as mean ± SEM (*n *= 10). One-way analysis of variance was performed followed with post-hoc Tukey’s test. ^*^P < 0.05, ^**^P < 0.01. CON, normal fiber level group; NC, dietary polysaccharides deprivation group; APSI, dietary polysaccharides deprivation diet with APS at 5 g/kg group, APSII, dietary polysaccharides deprivation diet with APS at 10 g/kg group; APSIII, dietary polysaccharides deprivation diet with APS at 30 g/kg group

To determine whether APS improves intestinal barrier function and whether polysaccharides are critical for intestinal barrier function, we fed broilers a polysaccharide deprivation diet (Fig. 1). Compared with normal feeds, the body weight and average daily gain of broilers on dietary polysaccharide deprivation were lower at 21 d, but the body weight and average daily gain of broilers were significantly higher in the APSI and APSIII groups than in the NC group at 21 d (*P* < 0.05, Fig. 3). In addition, the feed conversion ratio of broilers was significantly higher in NC groups than that in CON groups at 21 d, but the feed conversion ratio of broilers was significantly lower in APSI and APSIII groups than that in NC group at 21 d (*P* < 0.05, Fig. 3).

### Positive effects of APS are based on the enhancement of the intestinal barrier function

**Fig. 4:**
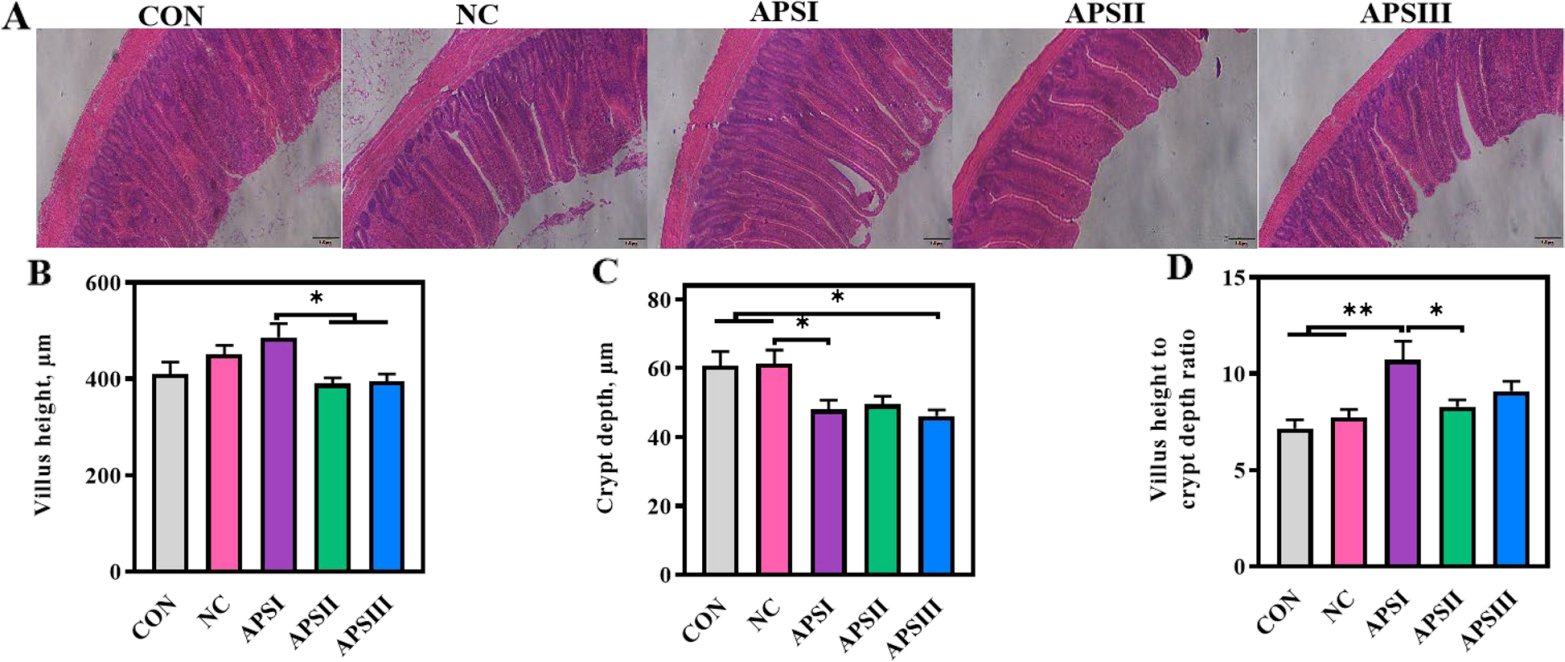
Effects of APS supplementation on the ileal morphology. **A** Representative images of H&E-stained ileal sections (100×). **B** Villus height. **C** Crypt depth. **D** Villus height to crypt depth ratio. All data are expressed as mean ± SEM (*n *= 10). One-way analysis of variance was performed followed with post-hoc Tukey’s test. ^*^P < 0.05, ^**^P < 0.01. CON, normal fiber level group; NC, dietary polysaccharides deprivation group; APSI, dietary polysaccharides deprivation diet with APS at 5 g/kg group; APSII, dietary polysaccharides deprivation diet with APS at 10 g/kg group; APSIII, dietary polysaccharides deprivation diet with APS at 30 g/kg group

Intestinal villi and crypts are key indicators of nutrient absorption capacity, and intestinal morphology is widely used to evaluate the intestinal barrier function. Compared with CON group, fiber deficiency did not affect intestinal villus height. Also, compared with the control group, the addition of APS did not cause changes in villus height, but compared with the APSI group, the APSII and APSIII groups significantly reduced villus height. Our results showed that the APSI and APSIII groups had a significantly reduced crypt depth of the ileum compared to the NC group at 21 d (*P* < 0.05; Fig. 4C). Furthermore, the APSI group had a significantly increased villus height to crypt depth ratio compared to the NC group at 21 d (*P* < 0.01, Fig. 4D). However, compared with the NC group, APS treatment did not significantly affect ileal villus height in broiler chickens (Fig. 4B).

**Fig. 5:**
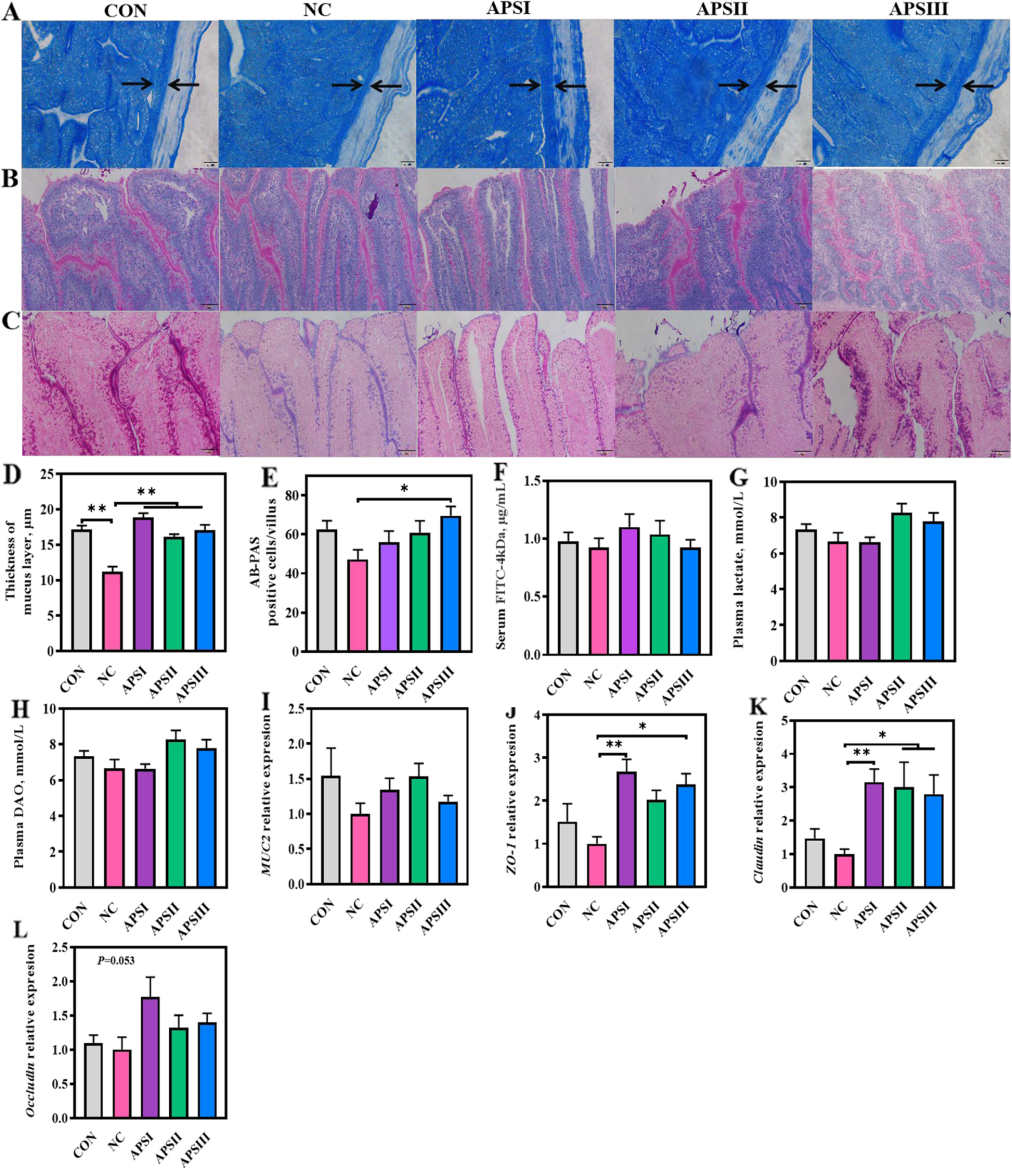
APS enhances intestinal barrier function. **A** and **D** Representative images of Alcian blue-stained ileal sections showing the thickness of mucus layer (400×). **B** Representative images of PAS-stained ileal sections (200×). **C** and **E** Representative images of AB-PAS-stained ileal sections showing the number of mucin (200×). **F**–**H** Intestinal permeability serum FITC-dextran levels, plasma lactic acid levels, and plasma diamine oxidase activities, respectively. **I**–**L** The mRNA levels of barrier-related gene. All data are expressed as mean ± SEM (*n *= 10). One-way analysis of variance was performed followed with post-hoc Tukey’s test. ^*^P < 0.05, ^**^P < 0.01. CON, normal fiber level group; NC, dietary polysaccharides deprivation group; APSI, dietary polysaccharides deprivation diet with APS at 5 g/kg group; APSII, dietary polysaccharides deprivation diet with APS at 10 g/kg group; APSIII, dietary polysaccharides deprivation diet with APS at 30 g/kg group; FITC, Fluorescein isothiocyanate; DAO, Diamine oxidase; Muc2, Mucin 2; ZO-1, Zonula occluden 1

Next, we investigated whether APS plays a regulatory role in intestinal barrier function. As shown in Fig. 5A–E, compared with the CON group, intestinal mucus layer injury was observed in the NC group. Notably, APS supplementation significantly increased the thickness of the ileal mucus layer (*P* < 0.01; Fig. 5A and D). Consistent with these results, the number of mucins was significantly higher in the APSIII group than in the NC group (*P* < 0.05, Fig. 5B, C, and E). However, the 3 key indicators of intestinal permeability, including serum FITC-d levels, plasma lactic acid levels, and plasma diamine oxidase activity, were not significantly different between the groups (Fig. 5F–H). The levels of tight junction protein-related genes, including *ZO-1* and *Claudin,* were higher in both APS groups than in the NC group (*P* < 0.05, Fig. 5J and K); however, the expression of *Muc2 and* *Occludin* showed no significant differences among all groups (Fig. 5I and L). These findings revealed that APS plays a critical role in enhancing intestinal barrier function in broiler chickens and can improve intestinal barrier dysfunction induced by dietary polysaccharide deprivation.

### The landscape of gut microbiota shaped by APS

**Fig. 6:**
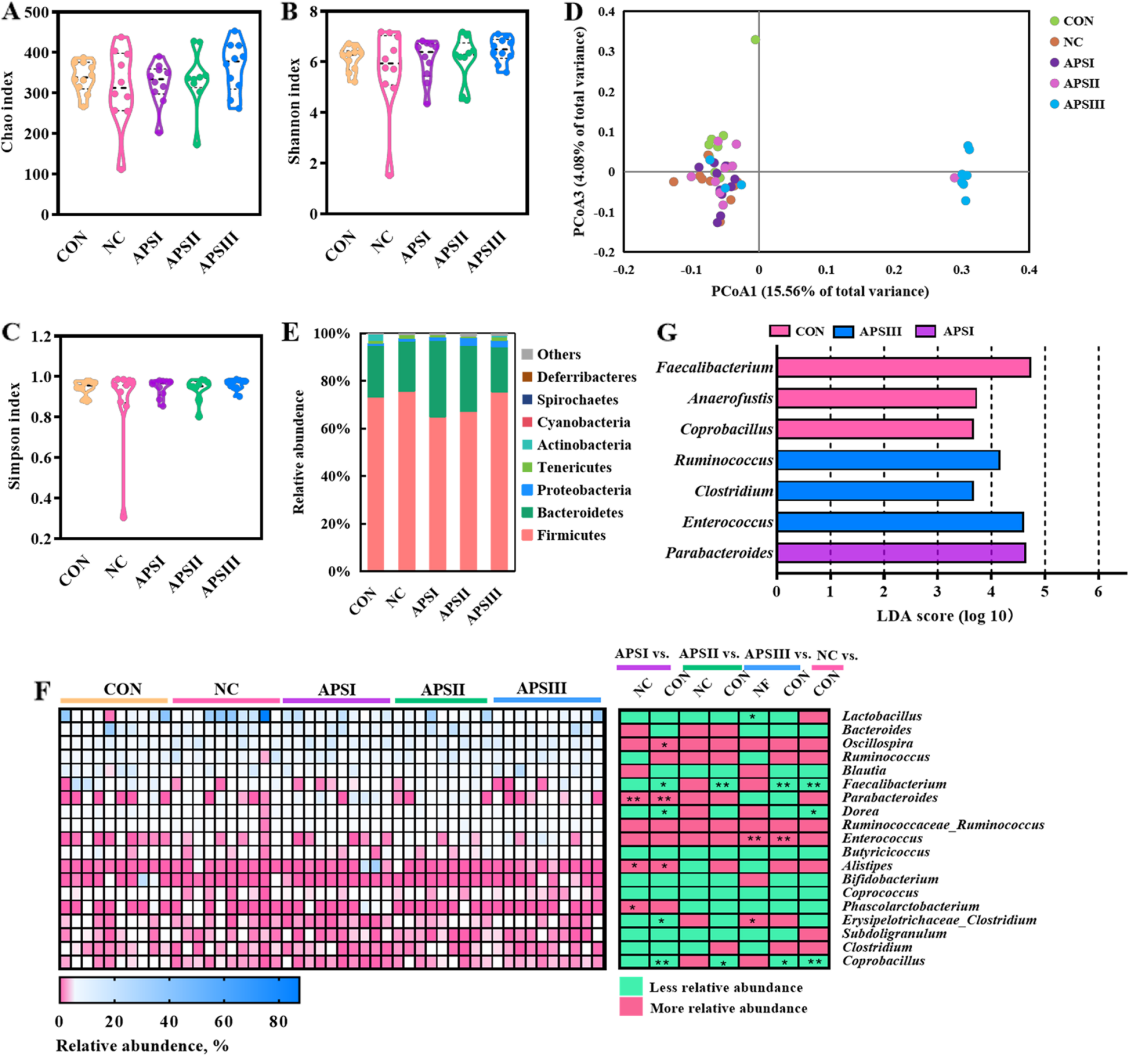
The cecum microbiota profile shaped by APS. **A–**C Alpha diversity analysis of gut microbiota, (**A**) Chao index, (**B**) Shannon index, (**C**) Simpson index. **D** Principal coordinate analysis (PCoA) of gut bacterial beta diversity based on Bray-Unifrac distance. **E** Relative abundance of bacteria at phylum. **F** Relative abundance of top 19 genera. **G** The most differential taxa at genus level were exhibited by LEfSe analysis. ^*^P < 0.05, ^**^P < 0.01. CON, normal fiber level group; NC, dietary polysaccharides deprivation group; APSI, dietary polysaccharides deprivation diet with APS at 5 g/kg group; APSII, dietary polysaccharides deprivation diet with APS at 10 g/kg group; APSIII, dietary polysaccharides deprivation diet with APS at 30 g/kg group

Our central hypothesis was that the gut microbiota acts as a bridge between dietary polysaccharides and gut health crosstalk [37]. Given that APS increases the thickness of the mucus layer and expression of tight junction protein-related genes, we examined whether the beneficial effects of APS are mediated by the gut microbiota. There was no significant difference in α-diversity of cecal microbiota including Chao index, Shannon index, and Simpson index (Fig. 6A–C). The community structure of the luminal microbiota in the cecum investigated by UniFrac-based principal coordinates analysis (PCoA) showed an obvious difference between the APSIII group and other groups at the OTU level (PERMANOVA, *P* < 0.05, Fig. 6D).

The composition of cecal microbiota in the five treatment groups is shown in Fig. 6E–G. At the phylum level, Firmicutes, Bacteroidetes, and Proteobacteria were predominant (Fig. 6E). At the genus level, the ceca of all groups harbored a high abundance of *Lactobacillus*, *Bacteroides*, *Oscillospira*, *Ruminococcus,* and *Blautia* (Fig. 6F). Compared to the CON group, the relative abundances of *Faecalibacterium*, *Dorea*, and *Coprobacillus* were lower in the NC group. The results further showed that the relative abundances of *Parabacteroides*, *Alistipes*, and *Phascolarctobacterium* were higher in the APSI group than in the control group (*P* < 0.05; Fig. 6F). In addition, the relative abundances of *Enterococcus* and *Erysipelotrichaceae_Clostridium* in the APSIII group were higher than those in the NC group (*P* < 0.05; Fig. 6F). However, the relative abundance of *Lactobacillus* in the APSIII group was lower than that in the control group (*P* < 0.05; Fig. 6F).

Further LEfSe analysis indicated that the cecal microbiota in the CON group was mainly enriched in genera such as *Faecalibacterium, Anaerofustis,* and *Coprobacillus* (Fig. 6G). The APSIII group was significantly enriched in *Ruminococcus, Clostridium,* and *Enterococcus* in the cecum (Fig. 6G). The cecal microbiota of the APSI group was mainly enriched in *Parabacteroides* (Fig. 6G)*.* These results revealed that the APS-induced gut microbiota might mediate the enhancement of intestinal barrier function.

### Gut microbiota-derived SCFAs and bile acids as potential markers for APS facilitating intestinal barrier function

**Fig. 7:**
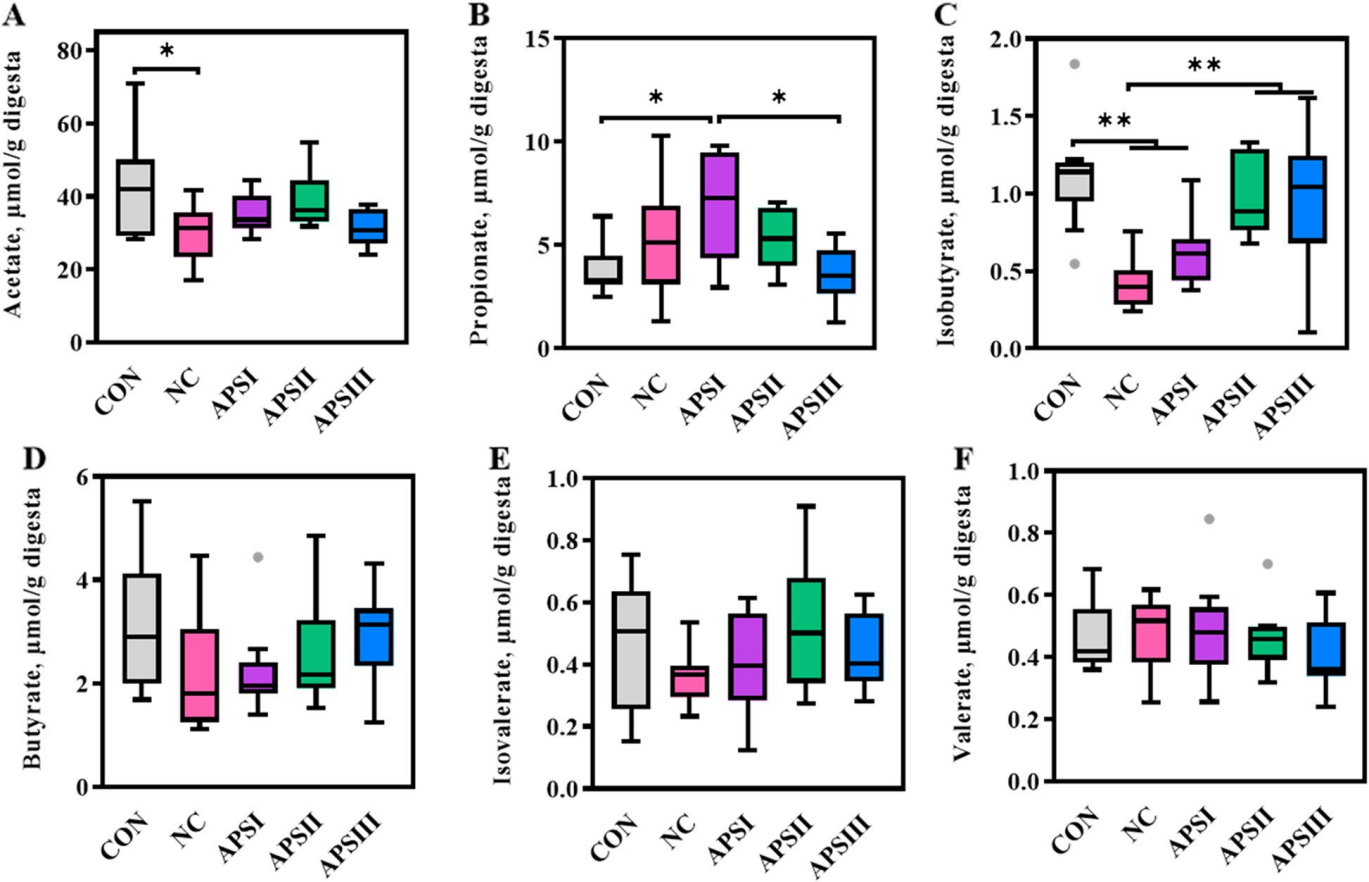
APS alters gut microbial SCFAs. Changes in cecal concentrations of (**A**) Acetate, (**B**) Propionate, (**C**) Isobutyrate, (**D**) Butyrate, (**E**) Isovalerate, and (**F**) valerate. All data are expressed as mean ± SEM (*n *= 10). One-way analysis of variance was performed followed with post-hoc Tukey’s test. ^*^P < 0.05, ^**^P < 0.01. CON, normal fiber level group; NC, dietary polysaccharides deprivation group; APSI, dietary polysaccharides deprivation diet with APS at 5 g/kg group; APSII, dietary polysaccharides deprivation diet with APS at 10 g/kg group; APSIII, dietary polysaccharides deprivation diet with APS at 30 g/kg group

Growing studies have demonstrated that gut microbiota-derived metabolites may be intermediates in the gut microbiota-intestinal barrier interaction [38]. Perturbation of the gut microbiota mediated by polysaccharides can cause a change in the metabolism of SCFAs, which are important mediators in the regulation of intestinal barrier function [12, 37]. The concentrations of cecal SCFAs in the five treatment groups are shown in Fig. 7. The concentration of isobutyrate in the APSII and APSIII groups was higher than that in the control group (*P* < 0.05; Fig. 7C). However, there were no significant differences in the levels of other SCFA, including acetate, propionate, butyrate, isovalerate, and valerate (Fig. 7).

**Fig. 8:**
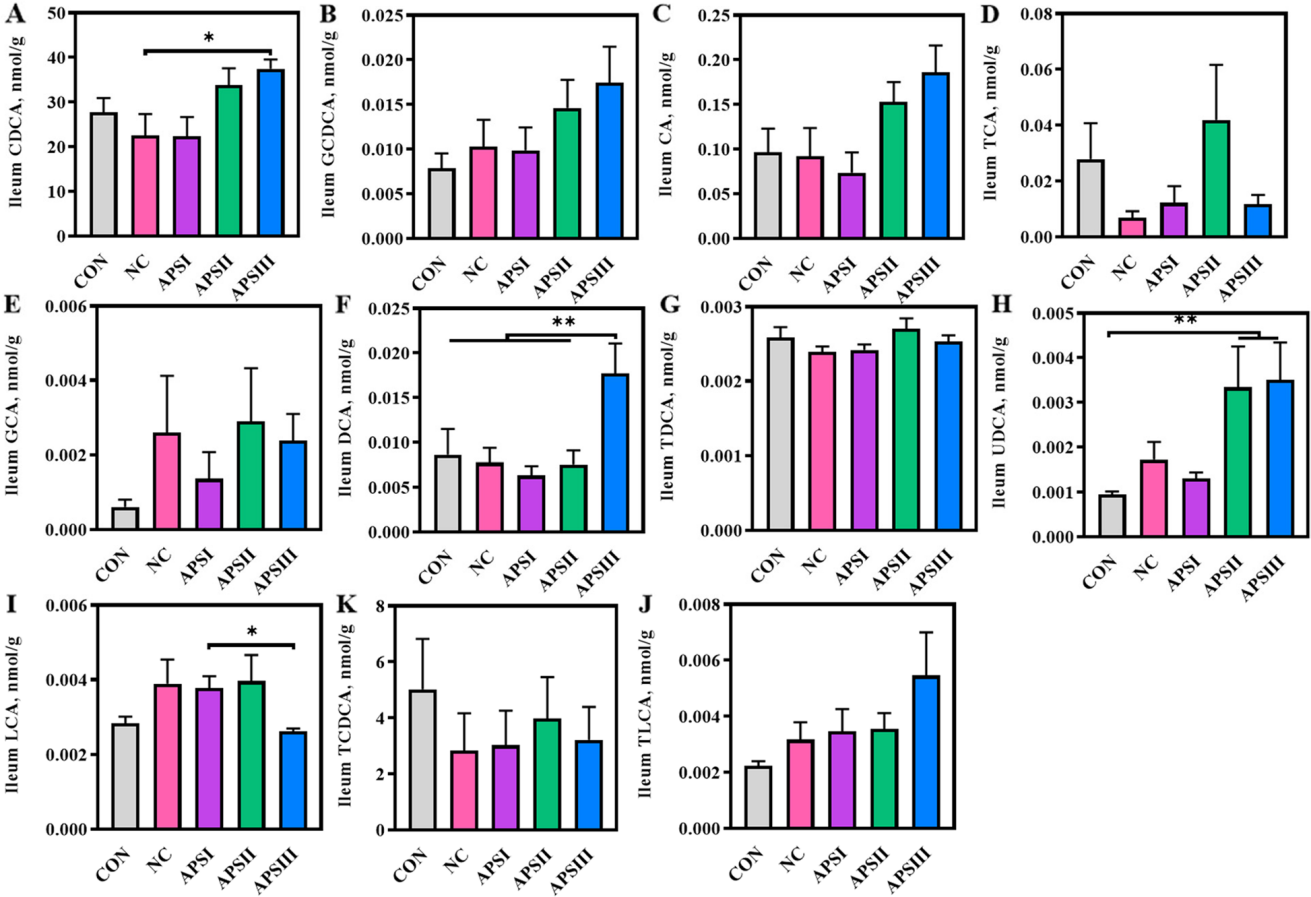
APS supplementation impacts microbial activities involved in bile acid biotransformation. The concentrations of (**A**) CDCA, (**B**) GCDCA, (**C**) CA, (**D**) TCA, (**E**) GCA, (**F**) DCA (**G**) TDCA, (**H**) UDCA, (**I**) LCA, (**J**) TLCA, and (**K**) TCDCA. All data are expressed as mean ± SEM (*n* = 10). One-way analysis of variance was performed followed with post-hoc Tukey’s test. ^*^P < 0.05, ^**^P < 0.01. CON, normal fiber level group; NC, dietary polysaccharides deprivation group; APSI, dietary polysaccharides deprivation diet with APS at 5 g/kg group; APSII, dietary polysaccharides deprivation diet with APS at 10 g/kg group; APSIII, dietary polysaccharides deprivation diet with APS at 30 g/kg group; CDCA, Chenodeoxycholic acid; GCDCA, Glycochenodeoxycholic acid; CA, Cholic acid; TCA, Taurocholic Acid; GCA, Glycocholic acid; DCA, Deoxycholate acid; TDCA, Taurodeoxycholic acid; UDCA, Ursodeoxycholic acid; LCA, Lithocholate; TLCA, Taurolithocholic acid; TCDCA, Taurochenodeoxycholic acid

With its strong bile salt hydrolase activity, the gut microbiota participates in bile acid metabolism through biotransformation [39]. Recent evidence suggests that altered gut microbiota composition by polysaccharides correlates with changes in bile acid composition and that bile acids can regulate intestinal barrier function [12, 40]. The concentrations of bile acids in the ileal contents are shown in Fig. 8; the concentrations of CDCA, deoxycholic acid (DCA), and ursodeoxycholic acid (UDCA) in the APSIII group were higher than those in the NC group (*P* < 0.05; Fig. 8A, F, and H). However, the APS-induced gut microbiota did not alter the concentration of bile acids in the plasma or liver (Fig. S1 and S2). These results suggest that gut microbiota-derived isobutyrate and bile acids are potential intermediates in APS that facilitate intestinal barrier function.

### An exclusive APS-induced microbiome signature

**Fig. 9:**
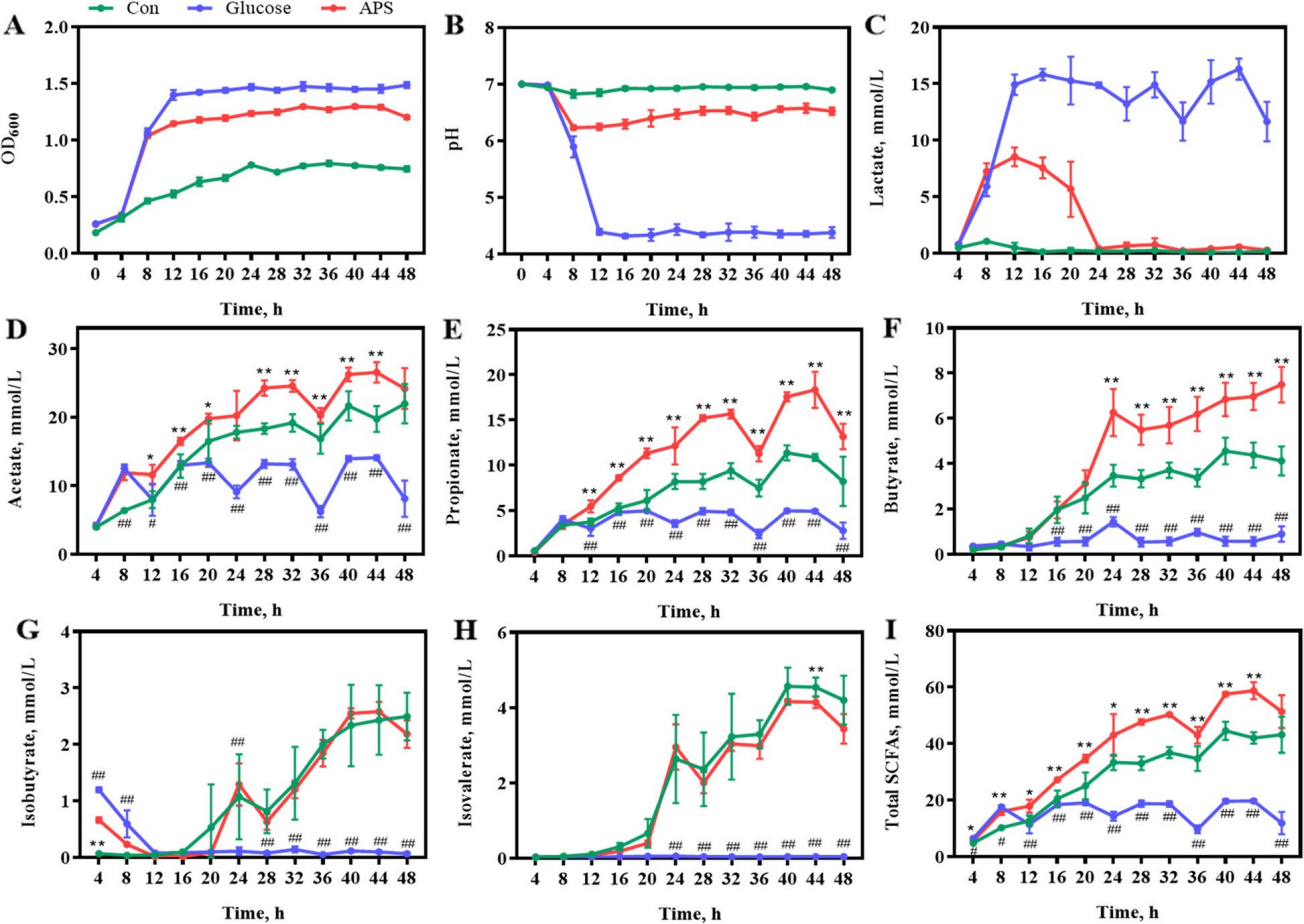
In vitro fermentation characteristics of APS. **A** Growth curves of cecal microbiota. **B–H **Change in supernatant concentrations of (**B**) pH, (**C**) Lactate, (**D**) Acetate, (**E**) Propionate, (**F**) Butyrate, (**G**) Isobutyrate, (**H**) Isovalerate. **F** Total SCFAs. All data are expressed as mean ± SEM (*n* = 5). ^*^P < 0.05, ^**^P < 0.01. Con, the medium without any carbon source group; Glucose, the medium containing 1% (w/v) glucose group; APS, the medium containing 1% (w/v) APS group

Polysaccharide foraging manipulates the microbial community and shapes the gut ecosystem. To confirm that APS can act as an energy source for microbial growth and alter the composition of the gut microbial community, we performed in vitro cecal microbial culture experiments using APS as the only carbohydrate source in the YCFA medium. The results showed that cecal microbiota grew efficiently with APS as the sole carbon source (Fig. 9). APS significantly reduces the pH of the medium, causing concomitant acidification of the medium (*P* < 0.05; Fig. 9). Furthermore, lactate and SCFAs (acetate, propionate, butyrate, isobutyrate, and isovalerate) in the medium changed during APS fermentation (Fig. 9). However, in vitro simulations of the host digestion data, including saliva, stomach, and small intestinal digestion, showed that the host could not degrade APS (Table 4).

**Table 4:** Simulated results of APS digestion in vitro

	mg/mL	0 h	1 h	2 h	4 h	*P*-value
CR	Saliva digestion	0.301 ± 0.00	0.317 ± 0.00	0.312 ± 0.00	0.321 ± 0.00	0.125
	Stomach digestion	0.798 ± 0.16	0.821 ± 0.03	0.835 ± 0.29	0.859 ± 0.62	0.169
	Small intestinal digestion	0.666 ± 0.00	0.665 ± 0.01	0.675 ± 0.01	0.699 ± 0.00	0.209
CT	Saliva digestion	0.855 ± 0.01	0.885 ± 0.00	0.862 ± 0.01	0.89 ± 0.013	0.216
	Stomach digestion	0.864 ± 0.00	0.862 ± 0.01	0.884 ± 0.02	0.892 ± 0.00	0.404
	Small intestinal digestion	0.564 ± 0.00	0.559 ± 0.00	0.583 ± 0.01	0.557 ± 0.00	0.217

*CR* Reducing sugar, *CT* Total sugar

**Fig. 10:**
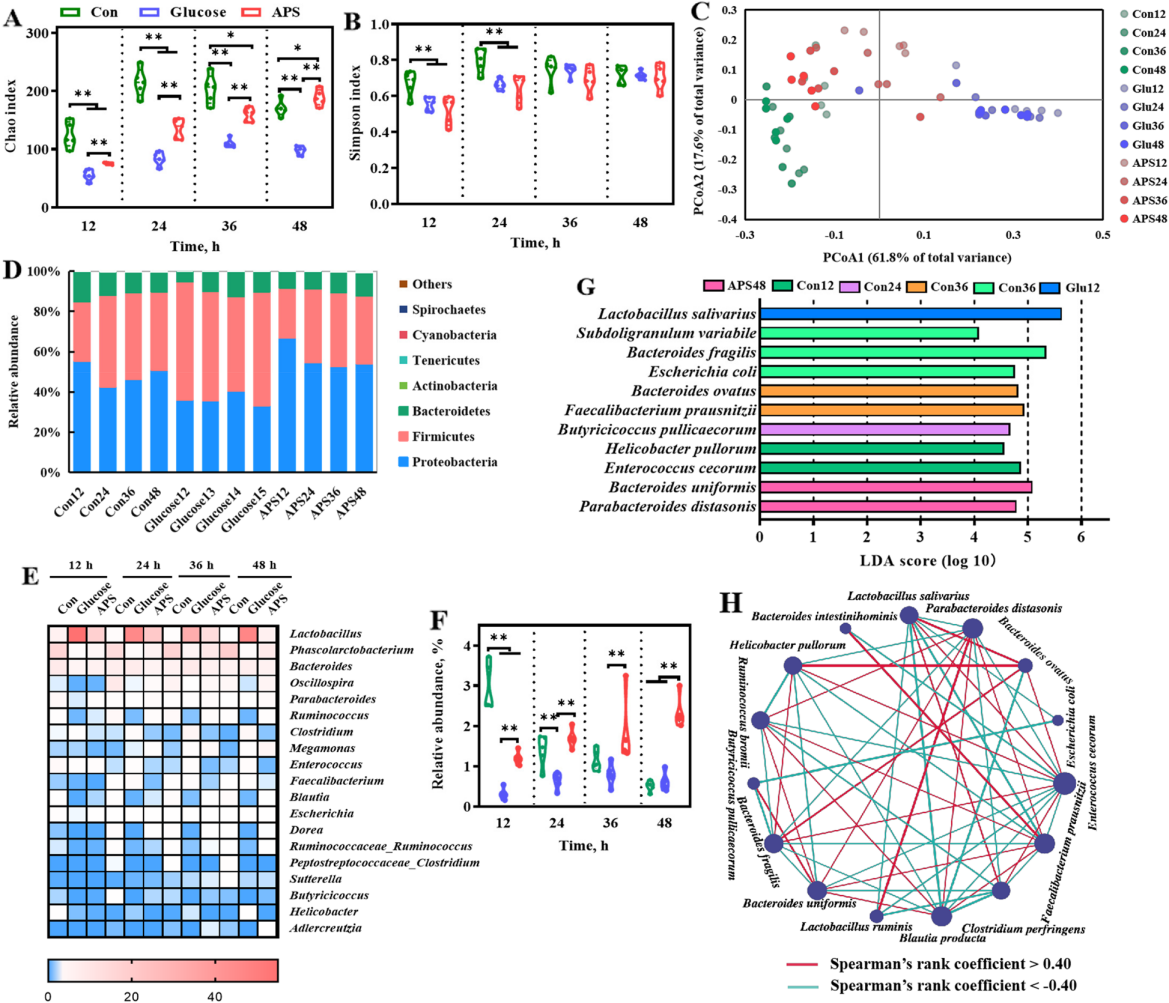
The APS-induced microbiome signature. Alpha diversity analysis of gut microbiota: (**A**) Chao index and (**B**) Simpson index. **C** Principal coordinate analysis (PCoA) of gut bacterial beta diversity based on Bray-Unifrac distance. **D** Relative abundance of bacteria at phylum. **E** Relative abundance of top 19 genera. **F** The change of *Parabacteroides*. **G** The most differential taxa at genus level were exhibited by LEfSe analysis. **H** Microbial co-occurrence network analysis based on species levels, the size of nodes was proportional to the relative abundance. All data are expressed as mean ± SEM (*n *= 5). ^*^P < 0.05, ^**^P < 0.01. Con, the medium without any carbon source group; Glucose, the medium containing 1% (w/v) glucose group; APS, the medium containing 1% (w/v) APS group

We further analyzed the APS-induced microbiota signatures in the medium. Compared with Con group, the α-diversity of APS group including Chao index and Simpson index were decreased (Fig. 10A and B). The results of the PCoA showed an obvious difference in the community structure among the Con, APS, and glucose groups at the OTU level (Fig. 10C). Consistent with the changes in beta diversity, the relative abundance of Firmicutes decreased in the APS group (Fig. 10D). At the genus level, *Lactobacillus*, *Phascolarctobacterium*, *Bacteroides*, *Oscillospira*, *Parabacteroides* were the dominant genus (Fig. 10E). Consistent with the results of dietary supplementation with APS on the intestinal microbiota in broiler chickens, the results of in vitro microbial culture showed that APS enriched the growth of *Parabacteroides* (Fig. 10F)*.* Further LEfSe analysis (the LDA score threshold was set at 4.0) indicated *Parabacteroides distasonis* and *Bacteroides uniformis* were mainly enriched in APS (Fig. 10G). Potential interactions between bacterial communities in response to APS were assessed using co-occurrence network analysis. The results showed that 19 species were correlated with each other and formed a large co-occurrence network during the degradation of APS (Fig. 10H). Interestingly, APS-enriched *Parabacteroides distasonis* and *Bacteroides uniformis* were positively correlated (Fig. 10H). Additionally, *Bacteroides uniformis* displays broad plasticity in the breakdown and uptake of polysaccharides [41]. These results suggest that APS is an important carbon source for the growth of gut microbes, which may be degraded by the gut commensal *Bacteroides uniformis* and shape the community structure (e.g., elevated abundance of *Parabacteroides distasonis*) through syntrophic interactions.

### APS alters the gene expression and activates the intestinal barrier function related signaling pathway

**Fig. 11:**
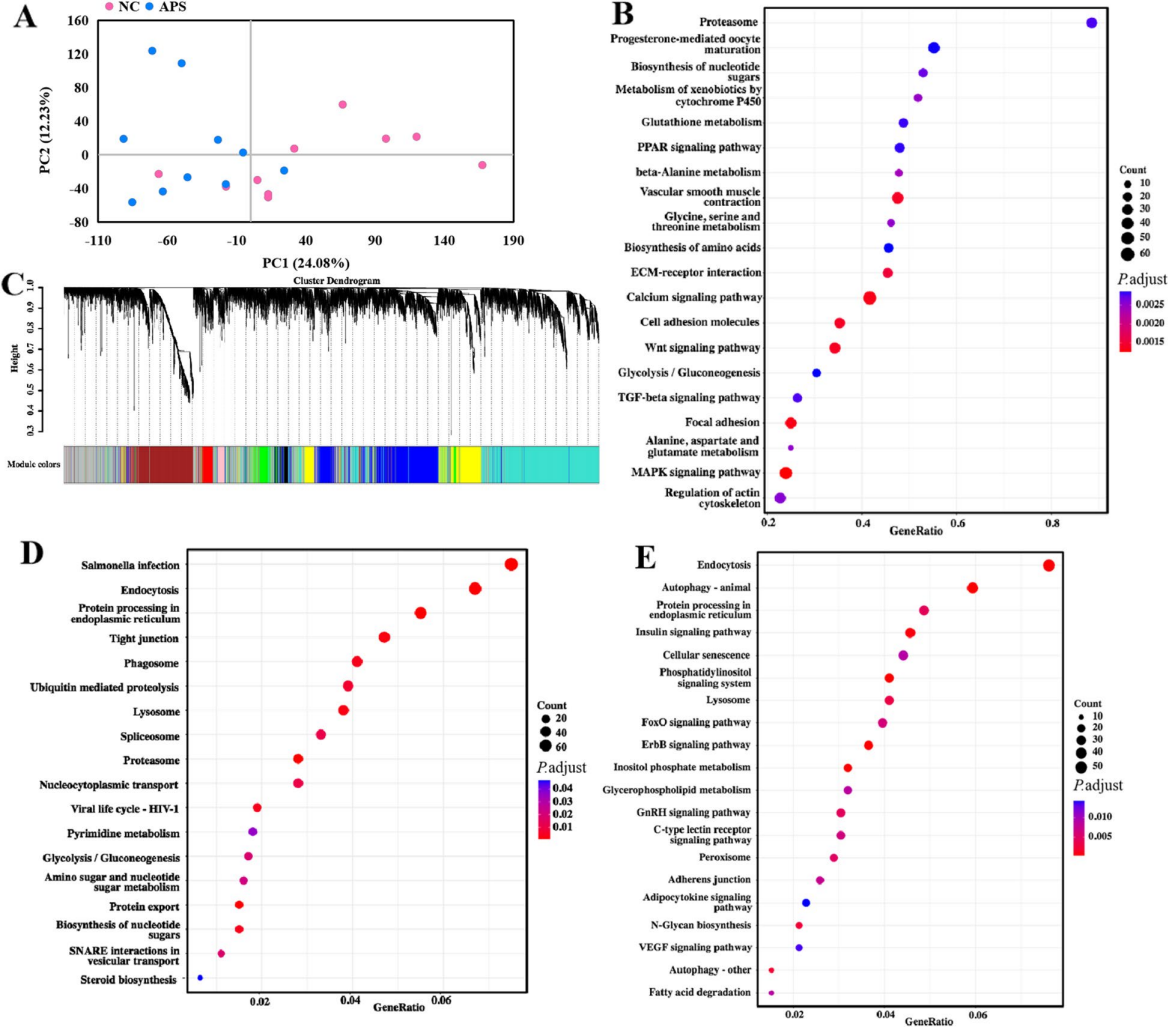
APS alters gene expression in ileum. **A** Principal component analysis (PCA) of transcriptional profiling. **B** Kyoto Encyclopedia of Genes and Genomes (KEGG) pathway enrichment analysis. **C** Division of gene modules. **D** and **E** KEGG pathway enrichment analysis of the key genes in turquoise modules and blue modules. All data are expressed as mean ± SEM (*n *= 5). ^*^P < 0.05, ^**^P < 0.01. NC, dietary polysaccharides deprivation group; APSIII, dietary polysaccharides deprivation diet with APS at 30 g/kg group

Our results showed that the APSIII group exhibited better effects in regulating the intestinal barrier function and gut microbiome. To further elucidate the potential mechanism underlying the enhancement of intestinal barrier function by APS-induced gut microbiota and microbiota-derived metabolites, we performed genome-wide transcriptional profiling of the ileal mucosa of the APSIII and NC groups for RNA sequencing. There were clear differences in the transcriptomes between the APS and NC groups (Fig. 11A). A total of 256 significantly altered genes were identified based on the significant false discovery rate *P* value criteria (fold change > 1 and *P* < 0.05). Among them, 131 genes were downregulated and 125 genes were upregulated in the ileum of the APS group compared to the NC group. Kyoto Encyclopedia of Genes and Genomes (KEGG) pathway analysis of differentially expressed genes revealed significant enrichment of signaling pathways such as the PPAR, Wnt, and MAPK signaling pathways, which may be involved in intestinal barrier function (Fig. 11B). Moreover, we calculated the adjacency and correlation matrices, combined them into a topology matrix, and a total of 9 gene modules (Fig. 11C). The turquoise and blue modules with the highest gene expression among all modules were selected for KEGG analysis. In the turquoise modules, protein processing in the endoplasmic reticulum and tight junction signaling pathways, which may be related to intestinal barrier function, were significantly enriched (Fig. 11D). In the blue modules, autophagy-animal, protein processing in the endoplasmic reticulum, adherens junction, and *N*-glycan biosynthesis signaling pathways, which may be related to intestinal barrier function, were significantly enriched (Fig. 11E).

## Discussion

Dysfunction of the intestinal barrier is associated with intestinal permeability, pathogen translocation, and systemic inflammatory responses [7, 42]. Although previous studies have shown that medicinal APS may play a key role in enhancing intestinal immunity and modulating the gut microbial ecosystem [28, 43], the APS-induced gut microbiota signature and protective mechanisms of intestinal barrier function have not been elucidated. In this study, we found that APS (especially at a 30 g/kg APS supplemental dose) obtained from *Astragalus membranaceus* water extract had beneficial effects on intestinal barrier dysfunction induced by dietary polysaccharide deprivation. Moreover, we demonstrated that the gut commensal *Bacteroides uniformis* might be required for the breakdown and uptake of APS, which in turn enriched the gut commensal *Parabacteroides* and increased the concentrations of isobutyrate and bile acids (mainly CDCA and DCA)*.* We found that intestinal barrier-related signaling pathways (such as protein processing in the endoplasmic reticulum, tight junctions, and adherens junction signaling pathways) have tremendous potential for protecting intestinal barrier function by APS-induced gut microbiota. These results suggest that the APS-induced gut microbiota play a predominant role in enhancing intestinal barrier function through intestinal barrier-related signaling activation.

The gut microbiota is a complex and dynamic ecosystem that plays a critical role in the maintenance of intestinal barrier function [37, 44]. Precise microbial community manipulation is recognized as an effective strategy for preventing gut dysfunction and sustaining current societal needs [45]. A growing body of evidence suggests that dietary polysaccharides regulate the gut microbiota and drive the formation of an exclusive metabolic niche, participating in host interactions [12, 46]. Previous study showed that the effect of APS on treating nonalcoholic fatty liver disease in high-fat diet-fed mice by enriching *Desulfovibrio vulgaris* [43, 47]. Another study of APS interaction with gut microbiota showed that APS attenuated immunosuppressive activity of myeloid-derived suppressor cells in melanoma-bearing mice by remodeling the gut microbiota and fecal metabolites [48]. Our previous studies revealed that APS has an immunomodulatory effect on the gut of broilers [27, 28]. In the present study, we found that APS enhanced the intestinal barrier function by manipulating the microbial community and enriching *Parabacteroides.* A similar study reported that the enrichment of *Parabacteroides* by polysaccharides isolated from the water of the medicinal fungus *Hirsutella sinensis* was associated with enhanced intestinal integrity and reduced levels of inflammation [49].

The breakdown and uptake of polysaccharides are mainly performed by intestinal bacteria based on various carbohydrate-active enzymes (CAZymes), such as *Bacteroides* and *Roseburia* [50, 51]. Complex polysaccharide foraging is typically facilitated by polysaccharide utilization loci (PULs), that encompass polysaccharide-binding proteins, CAZymes, and oligosaccharide transporter proteins [52]. *Bacteroides* dedicate approximately 18% of its genome to PULs and degrade a wide variety of dietary polysaccharides [37, 53]. Our data indicate that APS from the water extract of *Astragalus membranaceus* is a complex heteropolysaccharide and that the gut microbiota is required for the degradation of APS by in vitro digestion and in vitro growth assays. Furthermore, *Bacteroides uniformis*, a gut commensal species with excellent polysaccharide utilization ability [41, 54], is enriched in APS and may act as a primary degrader of APS. In recent years, an increasing number of studies have shown that myriad gut microbes exhibit highly intricate interactions and that cross-feeding mediated by polysaccharide foraging plays a crucial role in microbial collaboration [52, 55]. After a polysaccharide is released by the primary degrader, energy from sugars (such as oligo/monosaccharides and acetate) is harvested by other bacteria [56, 57]. In our study, the microbial co-occurrence network analysis showed a positive correlation between *Bacteroides uniformis* and *Parabacteroides distasonis*. These results imply that *Bacteroides uniformis* may degrade APS and enrich the colonization of *Parabacteroides distasonis* through cross-feeding, thereby improving intestinal barrier function.

Although previous studies have established the crucial function of gut microbiota in intestinal barrier function, illustrating that the potential mechanism that enhance intestinal barrier function remains urgently needed [12, 58]. Medicinal polysaccharides promote intestinal barrier function mainly via microbial community structure and metabolites, such as SCFAs, CDCA, and UDCA. [12, 24, 49]. It should be noted that the APS-induced gut microbiota increased the production of isobutyrate, CDCA, DCA, and UDCA. Previous studies have shown that changes in the gut microbiota caused by polysaccharides can alter the composition of SCFAs [12]. *Parabacteroides* plays a key role in the biotransformation of bile acids via bile salt hydrolases [59, 60]. These results suggest that isobutyrate and bile acids promote intestinal barrier function in a gut microbiota-dependent manner. Notably, based on RNA sequencing analysis, we noted that intestinal barrier-related signaling pathways (such as protein processing in the endoplasmic reticulum, tight junctions, and adherens junction signaling pathways) were significantly enriched in the APS-induced gut microbiota. A previous study showed that protein processing in endoplasmic reticulum signaling pathways is associated with mucin production [61]. Tight junction and adherens junction signaling pathways are also associated with intestinal barrier function [62]. Nevertheless, further studies are required to determine whether the SCFASs/bile acids produced by the APS-induced gut microbiota regulate intestinal barrier-related signaling pathways.

## Conclusion

In conclusion, our results indicate that APS (especially at 30 g/kg APS supplemental dose) from the extract of medicinal *Astragalus membranaceus* confers beneficial effects on dietary polysaccharide deprivation-induced intestinal barrier dysfunction by modulating microbial community composition. Our findings show that APS may be degraded by *Bacteroides uniformis* as a prebiotic when the gut commensal *Parabacteroides* contributes to intestinal barrier function. In addition, APS-induced gut microbiota-derived isobutyrate and bile acids (CDCA, DCA, and UDCA) may serve as potential biomarkers for intestinal barrier maintenance by activating intestinal barrier-related signaling pathways. Our findings highlight that APS-induced gut microbiota is a potential avenue for protecting intestinal barrier function.

### Supplementary Information

**Additional file 1**: **Fig S1**. Effect of APS supplementation on bile acids of plasma. The concentrations of (**A**) CDCA, (**B**) GCDCA, (**C**) CA, (**D**) TCA, (**E**) GCA, (**F**) DCA, (**G**) TDCA, (**H**) UDCA, (**I**) LCA, (**J**) TLCA, and (**K**) TCDCA. All data are expressed as mean ± SEM (*n* = 10). One-way analysis of variance was performed followed with post-hoc Tukey’s test. ^*^P < 0.05, ^**^P < 0.01. **Fig S2**. Effect of APS supplementation on bile acids of liver. The concentrations of (**A**) CDCA, (**B**) GCDCA, (**C**) CA, (**D**) TCA, (**E**) GCA, (**F**) DCA, (**G**) TDCA, (**H**) UDCA, (**I**) LCA, (**J**) TLCA, and (**K**) TCDCA. All data are expressed as mean ± SEM (*n* = 10). One-way analysis of variance was performed followed with post-hoc Tukey’s test. ^*^P < 0.05, ^**^P < 0.01.

## Data Availability

The data produced or analyzed during the current study are available from the corresponding author by reasonable request.

## Abbreviations

APS: *Astragalus* polysaccharides
CA: Cholic acid
CDCA: Chenodeoxycholic acid
CAZymes: Carbohydrate-active enzymes
DCA: Deoxycholate acid
FDR: False discovery rate
FITC: Fluorescein isothiocyanate
GCDCA: Glycochenodeoxycholic acid
KLF4: Kruppel-like factor 4
KEGG: Kyoto Encyclopedia of Genes and Genomes
LEfSe: Liner discriminant analysis effect size
LCA: Lithocholate
Muc2: Mucin 2
MAPK: Mitogen-activated protein kinase
PCoA: Principal coordinate analysis
PPAR: Peroxisome proliferators-activated receptor
PUL: Polysaccharide utilization loci
SCFAs: Short-chain fatty acids
TCA: Taurocholic acid
GCA: Glycocholic acid
TLCA: Taurolithocholic acid
TCDCA: Taurochenodeoxycholic acid
TDCA: Taurodeoxycholic acid
UDCA: Ursodeoxycholic acid
ZO-1: Zonula occluden 1
