# Supplementary material for: Astragalus polysaccharides-induced gut microbiota play a predominant role in enhancing of intestinal barrier function of broiler chickens
Source: J Anim Sci Biotechnol. 2024 Aug 6;15:106. doi: 10.1186/s40104-024-01060-1 (PMC11302362; doi:10.1186/s40104-024-01060-1)
Supplement: Supplementary file 1 — Additional file 1: Fig S1. Effect of APS supplementation on bile acids of plasma. The concentrations of (A) CDCA, (B) GCDCA, (C) CA, (D) TCA, (E) GCA, (F) DCA, (G) TDCA, (H) UDCA, (I) LCA, (J) TLCA, and (K) TCDCA. All data are expressed as mean ± SEM (n = 10). One-way analysis of variance was performed followed with post-hoc Tukey’s test. *P < 0.05, **P < 0.01. Fig S2. Effect of APS supplementation on bile acids of liver. The concentrations of (A) CDCA, (B) GCDCA, (C) CA, (D) TCA, (E) GCA, (F) DCA, (G) TDCA, (H) UDCA, (I) LCA, (J) TLCA, and (K) TCDCA. All data are expressed as mean ± SEM (n = 10). One-way analysis of variance was performed followed with post-hoc Tukey’s test. *P < 0.05, **P < 0.01. [file 40104_2024_1060_MOESM1_ESM.docx]

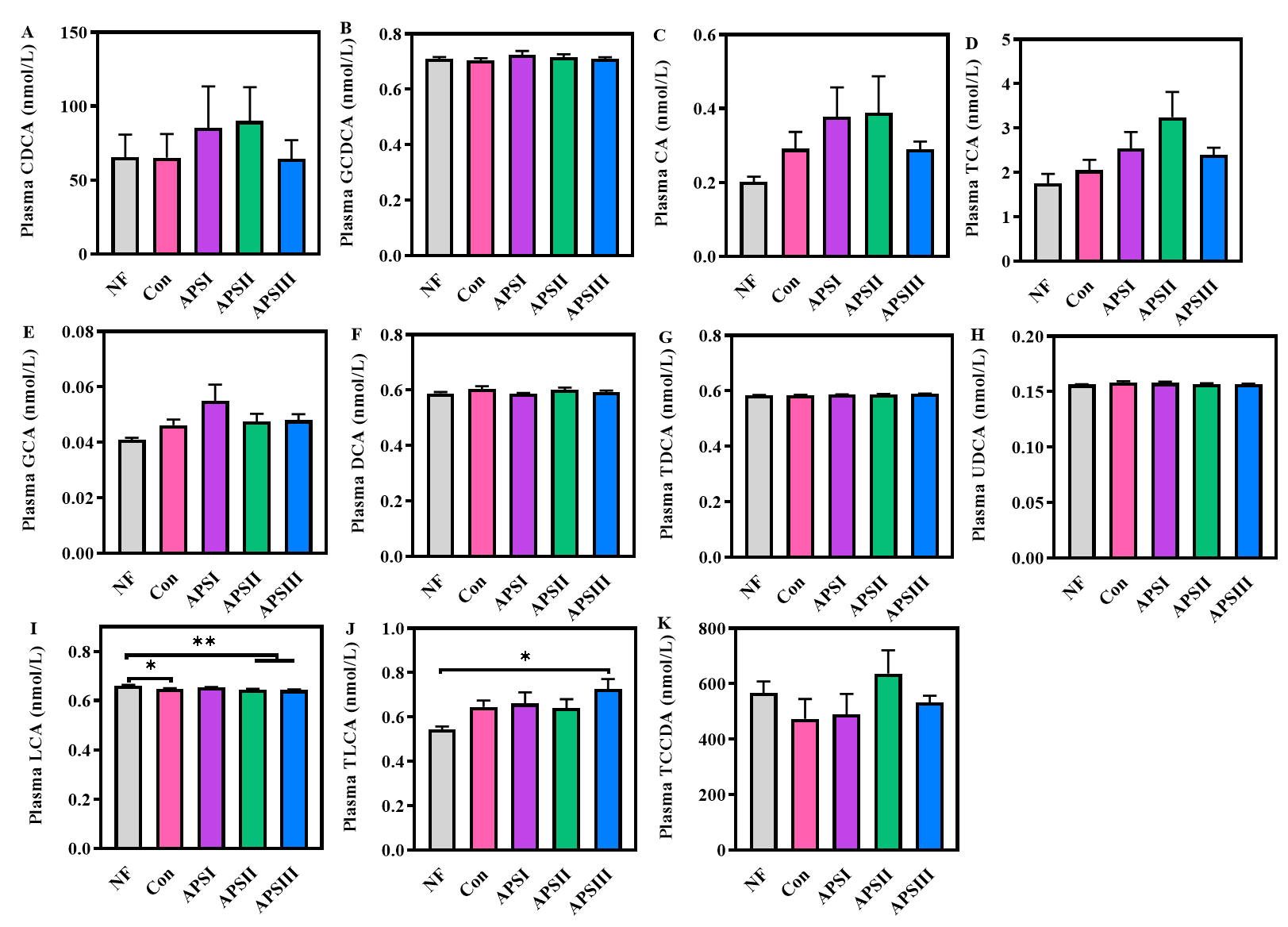
**Fig. S1** Effect of APS supplementation on bile acids of plasma. The concentrations of (**A**) CDCA, (**B**) GCDCA, (**C**) CA, (**D**) TCA, (**E**) GCA, (**F**) DCA (**G**) TDCA, (**H**) UDCA, (**I**) LCA, (**J**) TLCA and (**K**) TCDCA. All data are expressed as mean ± SEM (*n* = 10). One-way analysis of variance was performed followed with post-hoc Tukey’s test. ^*^*P* < 0.05, ^**^*P* < 0.01


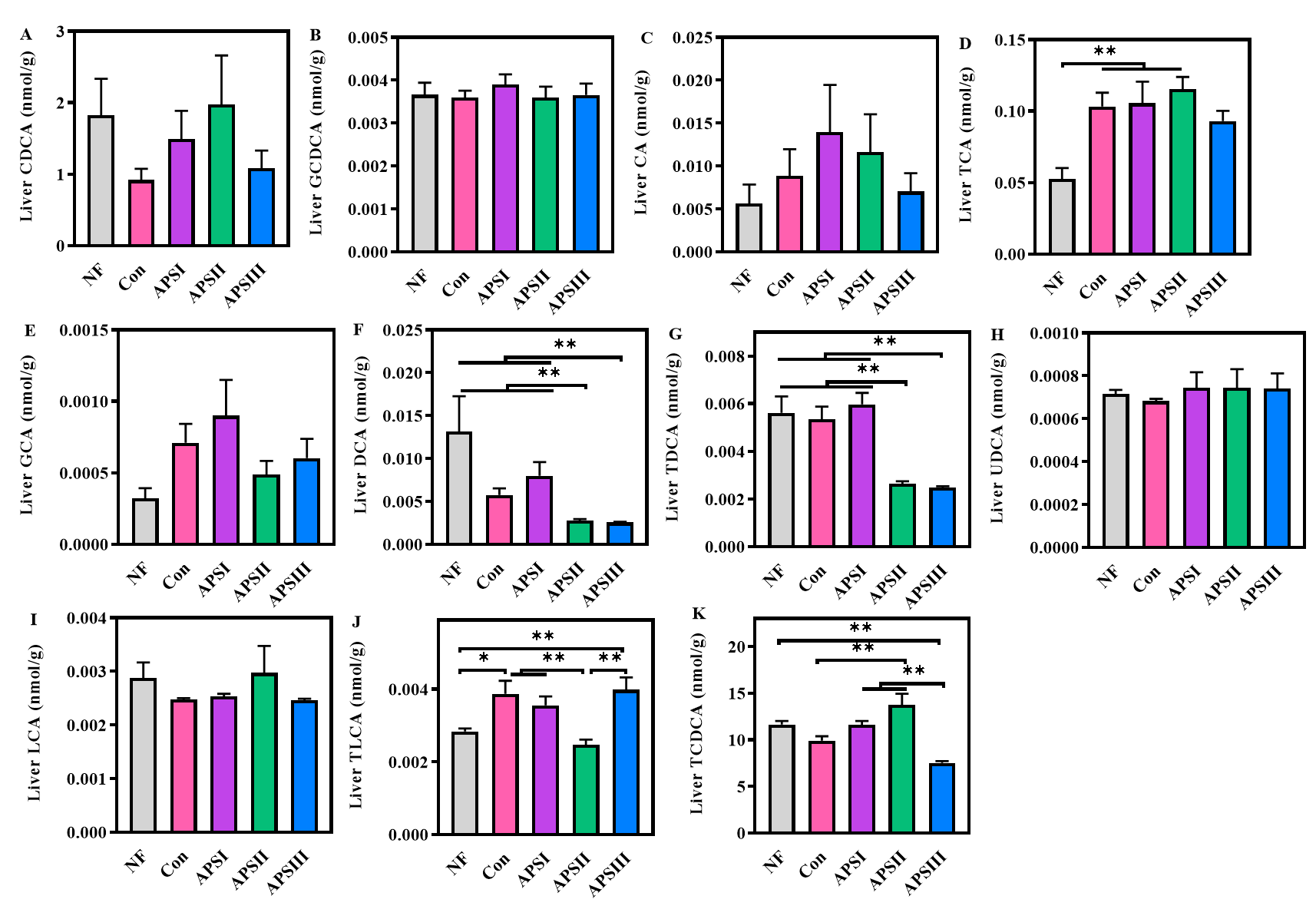


**Fig S2** Effect of APS supplementation on bile acids of liver. The concentrations of (**A**) CDCA, (**B**) GCDCA, (**C**) CA, (**D**) TCA, (**E**) GCA, (**F**) DCA, (**G**) TDCA, (**H**) UDCA, (**I**) LCA, (**J**) TLCA and (**K**) TCDCA. All data are expressed as mean ± SEM (*n* = 10). One-way analysis of variance was performed followed with post-hoc Tukey’s test. ^*^*P* < 0.05, ^**^*P* < 0.01
